# Intra-professional and inter-professional referral patterns of chiropractors

**DOI:** 10.1186/1746-1340-14-12

**Published:** 2006-07-06

**Authors:** Monica Smith, Barry R Greene, Mitchell Haas, Veerasathpurush Allareddy

**Affiliations:** 1 Palmer College of Chiropractic, Davenport, Iowa, USA, Palmer Center for Chiropractic Research, Davenport, Iowa, USA; 2 Department of Health Management and Policy, College of Public Health, The University of Iowa, Iowa City, Iowa, USA; 3 Western States Chiropractic College, Portland, Oregon, USA

## Abstract

**Background:**

With the increasing popularity of chiropractic care in the United States, inter-professional relationships between conventional trained physicians (MDs and DOs) and chiropractors (DCs) will have an expanding impact on patient care. The objectives of this study are to describe the intra-professional referral patterns amongst DCs, describe the inter-professional referral patterns between DCs and conventional trained medical primary care physicians (MDPCPs), and to identify provider characteristics that may affect these referral behaviors.

**Methods:**

A survey instrument to assess the attitudes and patterns of referral and consultation between MD primary care physicians (MDPCPs) and DCs was developed and sent to all DCs in the state of Iowa. Multivariable logistic regression models were built to assess the impact of provider characteristics on intra-professional and inter-professional referral patterns.

**Results:**

Of all DCs contacted, 452 (40.7%) participated in the study. Close to 8% of DCs reported that they never send a case report when referring a patient to another DC, while 13% never send a case report to a MDPCP. About 10% of DCs never send follow-up clinical information to referring doctors. DCs that perform differential diagnosis were significantly more likely to have engaged in inter-professional referral than DCs who did not perform differential diagnosis.

**Conclusion:**

The tendency toward informality, in both referral practices and sharing of clinical documentation for referred patients between MDPCPs and DCs, is an explicit marker of concerns that need to be addressed in order to improve coordination and continuity of care for patients shared between these provider types.

## Background

An increasing number of Americans are receiving health care services from alternate care providers [1-3]. Close to 42% of Americans received at least 1 of 16 alternate care therapies in 1997 and chiropractic care is one of the most frequently sought after alternative care [3]. With the increasing popularity of chiropractic care in the United States, inter-professional relationships between conventional trained physicians (MDs and DOs) and chiropractors (DCs) will have an expanding impact on patient care. Several studies have examined the attitudes of physicians towards alternate care therapies and alternate care providers [4-6]. There is an increasing body of evidence suggesting that poor inter-professional relationships between MDs and alternate care providers can lead to fragmentation of care and an eventual compromise in the quality of care delivered to patients [7]. While several studies of late have discussed the inter-professional relationships and referral patterns between MDs and alternate care providers from the perspective of MDs [8-11], only a few have examined the inter-professional referral patterns from the perspective of a DC [12,13].

The objectives of our study were to describe the intra-professional referral patterns amongst DCs, describe the inter-professional referral patterns between DCs and conventionally trained medical primary care physicians (MDPCPs), and to identify provider characteristics that may affect these referral behaviors. Toward these ends, we surveyed both MDPCPs and DCs in Iowa. We report here DC perspectives on professional relationships. The MDPCP survey findings have been published in a companion paper [14].

## Methods

We developed a pair of survey instruments to assess the attitudes and patterns of referral and consultation between MD primary care physicians (MDPCPs) and DCs. The survey instruments were modified based on feedback obtained from focus group interviews of MDPCPs and DCs, and from pilot testing of the survey instruments. The DC survey may be found in the Appendix (See additional file 1).

We mailed the survey to all DCs licensed in the state of Iowa, based on the list obtained from the Iowa Board of Chiropractic Examiners in 2001. We contacted by mail a total of 1,111 DCs and solicited their participation in this survey. A second mailing was sent to those who did not respond to the first mailing.

Descriptive statistics were used to examine the responses of DCs to the various questions about their intra-professional and inter-professional patterns of referrals, consults, and sharing of clinical information. Multivariable logistic regression was used to examine intra-professional (DC-to-DC) and inter-professional (DC-to-MDPCP) relationships. Separate models were developed for referral and consult outcomes of interest. We assessed the impact of three variables on referral/consult behaviors: age, sex, and whether the DC performed differential diagnosis. Age was divided into four categories: 26 – 35, 36 – 45, 46 – 55, and > 55 years of age (reference category). For sex, female was used as the reference category. DCs who performed differential diagnosis in their chiropractic examination and assessment of a patient's condition were compared to those DCs who only assessed their patients for "subluxation" (reference category), that is, segmental spinal lesion/dysfunction.

Logistic models were examined with the Hosmer and Lemeshow goodness of fit test. A two-tailed p-value of less than 0.05 was deemed to be statistically significant for all analyses. SAS version 9.1 and SPSS version 13.1 were used for statistical analyses.

## Results

A total of 452 DCs volunteered to participate in the study, for a survey response rate of 40.7%. This response rate is comparable to that obtained in other surveys of chiropractors [15]. We compared participants to non-participants using demographic data on age and sex that was available from the state licensure rosters, and found no significant differences. The mean age of the participants was 45 years. Participants included 313 men and 113 women. For 26 participants, data regarding sex was not available.

Tables 1 and 2 describe the intra-professional and inter-professional referral patterns of DCs. Approximately 74% of DCs have referred patients to other DCs for a health complaint, and the most common reasons for DC-to-DC referral were "seeking specific technique or expertise", "disability or impairment rating", and "second opinion". Almost 63% reported that they typically initiate a formal referral rather than have their patients contact the other DC on their own. Almost all of the DCs have recommended patients see an MD (99.8%). Similar to the intra-professional rate, approximately 57% of DCs recommended that they initiate a formal referral to MDs. Most DCs (91%) have formally referred a patient to an MD at some time. The most common health complaints for which DCs referred their patients to MDs were: cardiac conditions, infectious conditions, neurological lesions, and conditions that were unresponsive to manipulation.

**Table 1:** Intra-professional Relationships of Chiropractors

**Question**	**Response**	**N (%)**
**Have you recommended patient try seeing other DC for complains?**	Yes	385 (93.4)
	No	27 (6.6)
**Do you recommend patients contact doctor on own or initiate formal referral yourself?**	Patient contact doctor	134 (36.9)
	Doctor initiates referral	229 (63.1)
**Have you referred a patient to other DC for evaluation or treatment**	Yes	305 (73.7)
	No	109 (26.3)
**How often referral includes sending case report?**^¶^	Always	116 (41.9)
	Usually	74 (26.7)
	Sometimes	64 (23.1)
	Never	23 (8.3)
**How often referral includes sending X-Rays or X-Ray report?**^¶^	Always	130 (47.1)
	Usually	87 (31.5)
	Sometimes	43 (15.6)
	Never	16 (5.8)
**How often referral includes sending clinical records other than X-Rays?**^¶^	Always	100 (37.9)
	Usually	71 (26.9)
	Sometimes	73 (27.6)
	Never	20 (7.6)
**How often referral includes sending reason for referrals?**^¶^	Always	224 (84.8)
	Usually	27 (10.2)
	Sometimes	10 (3.8)
	Never	3 (1.1)
**Have you accepted referral from other doctors?**	Yes	320 (76.4)
	No	99 (23.6)
**How often do you send clinical information to referring doctor as follow-up to referral?**^¥^	Always	81 (25.9)
	Usually	91 (29.1)
	Sometimes	109 (34.8)
	Never	31 (9.9)
**Have you refused referral from a doctor?**	Yes	34 (8.2)
	No	381 (91.8)
**Has other DC obtained clinical information or advice via curbside consultation**	Yes	356 (84.6)
	No	65 (15.4)
**Have you obtained clinical information or advice from another DC via curbside consultation?**	Yes	342 (82.8)
	No	71 (17.2)

¶ – Questions are applicable for respondents who had referred a patient to another DC for evaluation or treatment.

¥ – Question is applicable for respondents who accepted a formal referral from a DC

**Table 2:** Inter-professional Relationships between Chiropractors and MDs

**Question**	**Response**	**N (%)**
**Have you ever recommended patient see a MD ?**	Yes	420 (99.8)
	No	1 (0.2)
**Do you recommend patient contacts MD on own or initiate formal referral ?**	Patient contacts MD	168 (43.4)
	Doctor initiates referral	219 (56.6)
**Have you ever referred patient to MD for evaluation or treatment?**	Yes	384 (91)
	No	38 (9)
**How often referral includes sending case report?**^¶^	Always	111 (31.2)
	Usually	90 (25.3)
	Sometimes	110 (30.9)
	Never	45 (12.6)
**How often referral includes sending X-rays or X-ray report?**^¶^	Always	103 (28.6)
	Usually	125 (34.7)
	Sometimes	106 (29.4)
	Never	26 (7)
**How often referral includes sending clinical records other than X-rays?**^¶^	Always	72 (21.1)
	Usually	84 (24.6)
	Sometimes	133 (38.9)
	Never	53 (15.5)
**How often referral includes sending reason for referral?**^¶^	Always	291 (82.7)
	Usually	45 (12.8)
	Sometimes	12 (3.4)
	Never	4 (1.1)
**Have accepted referral from a MD**	Yes	275 (66.3)
	No	140 (33.7)
**How often do you send clinical information to referring MD as follow-up to referral?**^¥^	Always	74 (27.2)
	Usually	68 (25)
	Sometimes	100 (36.8)
	Never	30 (11)
**Have refused a referral from a MD?**	Yes	17 (4)
	No	403 (96)
**Has a MD obtained clinical information or advice via curbside consultation**	Yes	129 (30.5)
	No	294 (69.5)
**Have you obtained clinical information or advice from a MD via curbside consultation?**	Yes	203 (48.4)
	No	216 (51.6)

¶ – Questions are applicable for respondents who had referred a patient to a MD for evaluation or treatment.

¥ – Question is applicable for respondents who accepted a formal referral from a MD

When referring a patient to an MD, 95.5% of DCs would always or usually send a reason for the referral. However, they were less inclined to send a full clinical report, with 43.5% stating that they only sometimes or never sent a formal case report when referring to an MD.

While 76% of DCs have accepted a referral from another DC, only 66% of DCs have accepted a referral from an MD. About 8% of respondents have refused a referral from another DC. The most common reasons were: considerations of scope of practice, belief that the patient could be better served by an MD, and fear of legal/malpractice litigations. Only 4% of DCs have refused a referral from an MD. The most common reasons were: the patient could be served well by another specialist, the patient was not a chiropractic case, and the patient had insurance issues.

With regards to informal consultation behaviors, most DCs (over 80%) had engaged in "curbside consultation" [16-20] with another DC. Only 48% of DCs had ever obtained information or advice from an MD via informal curbside consult, and only 30% of DCs had ever offered a curbside consult to an MD.

The results of the multivariable analyses predicting the intra-professional referral patterns of DCs are summarized in Table 3. DCs in the youngest age group (26 – 35 years) were significantly less likely to have refused a referral from another DC (OR = 0.22, 95% CI = 0.05 – 0.92) when compared to DCs in the oldest age group (>55 years). DCs in all 3 younger age groups were more likely to be involved in curbside consultation practices when compared to those in the oldest age group (P < 0.05). The sex of the DCs was not a significant predictor of intra-professional referral patterns.

**Table 3:** Predictors of Intra-professional Referral Patterns

**Predictors**	**Do you recommend initiating a formal referral with a DC?** **OR (95% CI)**	**Have you refused a referral from a DC?** **OR (95% CI)**	**Has a DC obtained clinical info or advice via curbside consultation?** **OR (95% CI)**	**Have you obtained clinical info or curbside consultation from a DC?** **OR (95% CI)**
**Age (in years)**				
26–35	0.79 (0.37 – 1.66)	0.22 (0.05 – 0.92) ^¥^	2.97 (1.17 – 7.52) ^¥^	5.02 (1.92 – 13.10) ^¥^
36–45	0.62 (0.31 – 1.22)	0.52 (0.19 – 1.43)	2.33 (1.03 – 5.22) ^¥^	2.16 (1.04 – 4.47) ^¥^
46–55	0.68 (0.35 – 1.35)	0.71 (0.28 – 1.82)	1.32 (0.63 – 2.76)	2.14 (1.04 – 4.39) ^¥^
>55*	1.00	1.00	1.00	1.00
**Sex**				
Male	0.76 (0.44 – 1.30)	1.04 (0.42 – 2.59)	1.17 (0.60 – 2.30)	0.88 (0.44 – 1.76)
Female*	1.00	1.00	1.00	1.00
**DC Type**				
Use ddx	1.36 (0.51 – 3.59)	1.83 (0.23 – 14.44)	1.39 (0.44 – 4.42)	1.15 (0.36 – 3.70)
No ddx*	1.00	1.00	1.00	1.00
**Number of Cases**	342	392	398	390
**Model Fit p-value**	0.89	0.97	0.39	0.37

* = Reference

¥ = Significant at p < 0.05

Use ddx = DCs who performed differential diagnosis in their chiropractic examination and assessment of a patient's condition.

No ddx = DCs who only assessed their patients for "subluxation", that is, segmental spinal lesion/dysfunction.

The results of the multivariable analyses predicting the inter-professional referral patterns between DCs and MDs are summarized in Table 4. DCs that perform differential diagnosis were significantly more likely to have engaged in inter-professional referral (OR = 4.5, 95% CI = 1.6 – 12.8) and made formal referrals (OR = 4.7, 95% CI = 1.7 – 13.0) than DCs who do not perform differential diagnosis. Neither age nor sex of DCs was a significant predictor of inter-professional referral patterns. However, DCs that perform differential diagnosis were significantly more likely to have engaged in inter-professional referral than DCs who do not.

**Table 4:** Predictors of Inter-professional Referral Patterns

**Predictors**	**Do you recommend initiating a formal referral with a MD?** **OR (95% CI)**	**Have you referred a patient to an MD for evaluation or treatment?** **OR (95% CI)**	**Has an MD obtained clinical info or advice via curbside consultation?** **OR (95% CI)**	**Have you obtained clinical info or curbside consultation from an MD?** **OR (95% CI)**
**Age (in years)**				
26–35	0.99 (0.50 – 1.98)	0.65 (0.20 – 2.07)	0.95 (0.46 – 1.92)	1.35 (0.70 – 2.61)
36–45	1.23 (0.64 – 2.35)	0.97 (0.30 – 3.13)	0.88 (0.45 – 1.70)	1.22 (0.66 – 2.26)
46–55	0.86 (0.46 – 1.63)	0.72 (0.23 – 2.22)	1.15 (0.60 – 2.18)	1.56 (0.85 – 2.87)
>55*	1.00	1.00	1.00	1.00
**Sex**				
Male	1.15 (0.69 – 1.91)	1.66 (0.77 – 3.61)	1.38 (0.82 – 2.34)	1.08 (0.67 – 1.72)
Female*	1.00	1.00	1.00	1.00
**DC Type**				
Use ddx	4.65 (1.66 – 12.99)^¥^	4.51 (1.59 – 12.75)^¥^	2.08 (0.68 – 6.34)	2.54 (0.96 – 6.70)
No ddx*	1.00	1.00	1.00	1.00
**Number of Cases**	365	399	400	397
**Model Fit p-value**	0.41	0.80	0.78	0.98

* = Reference

¥ = Significant at p < 0.05

Use ddx = DCs who performed differential diagnosis in their chiropractic examination and assessment of a patient's condition.

No ddx = DCs who only assessed their patients for "subluxation", that is, segmental spinal lesion/dysfunction.

## Discussion

Our study suggests that DCs tend to engage in informal practices when recommending or referring their chiropractic patients to the care of an MDPCP. This tendency toward informal "lay referrals" was revealed to be reciprocal in our companion survey of MDPCPs, which showed that MDPCPs were much more likely to suggest that their patients contact a chiropractor on their own rather than to initiate a formal referral [14]. The lack of a direct formalized referral relationship between DCs and MDPCPs has implications for efficiency, quality, and patient safety in the health care delivery system. For example, there is empirical evidence suggesting that allowing patients to contact other physicians on their own is likely to break continuity of care [7,21].

Results from another study that examined the attitudes of DCs concerning referral to other health care providers [13] showed that DCs most commonly referred to MD specialists such as orthopedic surgeons and neurologists, and that common reasons for making such referrals were "second opinion" or "legal" considerations such as personal injury claims and litigations. In that study, close to 70% of the DCs mentioned that they received requests for patient records from medical physicians, 88% submitted requests for patient records to medical offices, and 80% submitted requests for patient records to hospitals [13]. These results suggest that there is a significant amount of professional interaction over patients shared between DCs and specialist medical physicians, including requests for formal clinical documentation. Our surveys of primary care MDs and DCs suggest that even when formal inter-professional referrals do occur between them, the initial communication of pertinent clinical information such as a patient case report is typically absent. However, we did not specifically query the extent to which clinical documentation is requested or supplied at some later point in the inter-professional referral process.

This context further underscores the importance of our survey finding that DCs who perform differential diagnosis are more likely to engage in formal referral behaviors with MDPCPs. The necessity of conducting a differential diagnosis and fully documenting the patient workup is an established standard for chiropractic education and practice [22-25] and serves to enhance the quality and coordination of care and improve the overall efficiency of integrative cross-disciplinary care practices.

A study conducted by Hawk and Dusio [12] reported on the coordination and continuity of services between DCs and MD/DOs from the perspective of DCs. They showed that 78% of DCs referred their patients to an MD/DO and 50% of DCs referred their patients to another DC during the 3 months prior to participating in the survey [12]. About 47% of DCs sent a report to an MD whereas only 33% sent reports to another DC [12]. Our study results are similar to those reported by Hawk and Dusio almost a decade ago, and further highlights the entrenched nature of this ongoing issue of discontinuity and poor coordination of services between DCs and MD/DOs.

The results of our current survey of DCs and our companion survey of MDPCPs clearly demonstrate that the inter-professional relationship between them is not conducive for maintaining the continuity of care [14]. Close to 82% of MDPCPs mentioned that their patients evinced interest in chiropractic care and approximately 72% of MDPCPs reported that their patients asked to be referred to a DC. However, only two-thirds of MDPCPs had ever recommended their patients to a DC, and when doing so most MDPCPs (88%) preferred their patients take the initiative to contact the DC on their own [14]. Only 28% of MDPCPs have ever formally referred their patients to a DC for evaluation or treatment, whereas when engaging in intra-professional referrals, most MD-PCPs (99%) preferred to formally refer their patients. While reluctant to refer patients to DCs, the MDPCPs were as likely as DCs to accept inter-professional referrals [14].

The tendency toward informality in both referral practices and sharing of clinical documentation for referred patients between MDPCPs and DCs is an explicit marker of concerns that need to be addressed in order to improve coordination and continuity of care for patients shared between these provider types. A conscious professional judgment to place the patient in a care process which is not fully informed, or is discontinuous, is related to quality of care and may be related to patient safety. Equally problematic, with slightly more insidious implications, are the disparities in intra-professional vs. inter-professional informal "curbside consultation" practices. While most MDPCPs (95%) and DCs (80%) engaged in intra-professional curbside consults, generally less than 30% of either ever experienced such informal consulting inter-professionally. We can speculate that the most obvious reason for this lack of informal inter-professional dialogue is probably largely due to the residual and historic isolation of chiropractic from medical practice and the dearth of multidisciplinary practice opportunities that otherwise might facilitate such inter-professional communication. Opportunities to readily engage in informal ongoing dialogue such as curbside consults can implicitly standardize and improve practices of care within disciplines and between generalist and specialist practice. What should be fully appreciated, however, is that ready access to such collegial input also serves an important and implicit mentoring function between senior and junior clinicians. In a multidisciplinary setting, informal consults have additional potential for standardizing and better integrating the provision of care between and across disparate clinical disciplines such as chiropractic and medicine.

Considering the fact that chiropractic care is increasing in popularity, it is important that we identify facilitators and barriers to developing positive inter-professional relationships between MDPCPs and DCs. More research needs to be directed at better understanding the issues surrounding the coordination of care between DCs and MDPCPs. This should include an examination of educational interventions to improve the documentation and sharing of clinical information and thereby enhance cross-disciplinary standards of care.

Finally, a limitation of our study is the low response rate. Only 40.7% of DCs contacted volunteered to participate in our study. The low participation rate raises issues about the external validity of our study. However, we should note that external validity can still be achieved with fewer participants provided there are no major differences between participants and non-participants [26,27].

## Conclusion

The study provides an insight into the intra-professional and inter-professional referral patterns of DCs. DCs tend to engage in informal practices when recommending or referring their chiropractic patients to the care of an MDPCP. The lack of a direct formalized referral relationship between DCs and MDPCPs has implications for efficiency, quality, and patient safety in the health care delivery system. Future studies must focus on identifying facilitators and barriers to developing positive inter-professional referral relationships between DCs and MDPCPs.

## Competing interests

The author(s) declare that they have no competing interests.

## Funding

This research was made possible by funding by NIH-NCCAM – Project #AT-01-001 – Analysis of DC MDPCP Interprofessional Relationships. This investigation was conducted in a facility constructed with support from Research Facilities Improvement Grant Number C06 RR15433 from the National Center for Research Resources, National Institute of Health.

## References

[B1] Astin JA (1998). Why patients use alternative medicine: results of a national study. Jama.

[B2] Eisenberg DM, Kessler RC, Foster C, Norlock FE, Calkins DR, Delbanco TL (1993). Unconventional medicine in the United States. Prevalence, costs, and patterns of use. N Engl J Med.

[B3] Eisenberg DM, Davis RB, Ettner SL, Appel S, Wilkey S, Van Rompay M, Kessler RC (1998). Trends in alternative medicine use in the United States, 1990-1997: results of a follow-up national survey. Jama.

[B4] Berman BM, Singh BK, Lao L, Singh BB, Ferentz KS, Hartnoll SM (1995). Physicians' attitudes toward complementary or alternative medicine: a regional survey. J Am Board Fam Pract.

[B5] van Haselen RA, Reiber U, Nickel I, Jakob A, Fisher PA (2004). Providing Complementary and Alternative Medicine in primary care: the primary care workers' perspective. Complement Ther Med.

[B6] Verhoef MJ, Sutherland LR (1995). Alternative medicine and general practitioners. Opinions and behaviour. Can Fam Physician.

[B7] Mainous AG, Gill JM, Zoller JS, Wolman MG (2000). Fragmentation of patient care between chiropractors and family physicians. Arch Fam Med.

[B8] Easthope G, Tranter B, Gill G (2000). General practitioners' attitudes toward complementary therapies. Social Science and Medicine.

[B9] Goldszmidt M, Levitt C, Duarte-Franco E, Kaczorowski J (1995). Complementary health care services: a survey of general practitioners' views. Cmaj.

[B10] Sikand A, Laken M (1998). Pediatricians' experience with attitudes toward complementary/alternative medicine. Arch Pediatr Adolesc Med.

[B11] Coulter ID, Singh BB, Riley D, Der-Martirosian C (2005). Interprofessional referral patterns in an integrated medical system. J Manipulative Physiol Ther.

[B12] Hawk C, Dusio ME (1995). A survey of 492 U.S. chiropractors on primary care and prevention-related issues. J Manipulative Physiol Ther.

[B13] Sawyer CE, Bergmann TF, Good DW (1988). Attitudes and habits of chiropractors concerning referral to other health care providers. J Manipulative Physiol Ther.

[B14] Greene BR, Smith M, Allareddy V, Haas M (2006). Referral Patterns and Attitudes of Primary Care Physicians Towards Chiropractors. BMC Complement Altern Med.

[B15] Russell ML, Verhoef MJ, Injeyan HS, McMorland DG (2004). Response rates for surveys of chiropractors. J Manipulative Physiol Ther.

[B16] Bergus GR, Randall CS, Sinift SD, Rosenthal DM (2000). Does the structure of clinical questions affect the outcome of curbside consultations with specialty colleagues?. Arch Fam Med.

[B17] Golub RM (1998). Curbside consultations and the viaduct effect. Jama.

[B18] Keating NL, Zaslavsky AM, Ayanian JZ (1998). Physicians' experiences and beliefs regarding informal consultation. Jama.

[B19] Kuo D, Gifford DR, Stein MD (1998). Curbside consultation practices and attitudes among primary care physicians and medical subspecialists. Jama.

[B20] Schulte M, Mehler PS (1999). Promoting primary care-subspecialist interaction through curbside consultations. J Gen Intern Med.

[B21] Lee T, Pappius EM, Goldman L (1983). Impact of inter-physician communication on the effectiveness of medical consultations. Am J Med.

[B22] Patient Examination, Assessment and Diagnosis in Chiropractic  Clinical Educations. Proceedings from an International Conference  (co-sponsored by the World Federation of Chiropractic, Association of  Chiropractic Colleges, and US National Board of Chiropractic Examiners).

[B23] Greeley CO Federation of Chiropractic Licensing Boards. http://www.fclb.org.

[B24] Haldeman S, Chapman-Smith D, Petersen D (1993). Guidelines for Chiropractic Quality Assurance and Practice Parameters.

[B25] National Board of Chiropractic Examiners (2005). Job  Analysis of Chiropractic. http://www.nbce.org.

[B26] Babbie E (2004). The Practice of Social Research.

[B27] Singh B, Liu XD, Der-Martirosian C, Hardy M, Singh V, Shepard N, Gandhi S, Khorsan R (2005). A national probability survey of American Medical Association gynecologists and primary care physicians concerning menopause. Am J Obstet Gynecol.

